# Supplementary material for: Multilineage murine stem cells generate complex organoids to model distal lung development and disease
Source: EMBO J. 2020 Sep 28;39(21):e103476. doi: 10.15252/embj.2019103476 (PMC7604576; doi:10.15252/embj.2019103476)
Supplement: Supplementary file 1 — Appendix [file EMBJ-39-e103476-s001.pdf]

Table of contents

Appendix Figure S1. Initial culture of BASC with rMC primarily gives rise to BALO with a minor proportion of bronchiolospheres and alveolospheres formation

Appendix Figure S2. The BALO system resembles the cellular composition and organization of the bronchioalveolar compartment

Appendix Figure S3. Defined rMC subsets contribute to BALO formation

Appendix Figure S4. Tissue-resident macrophages engraft into BALO

Appendix Figure S5. Downregulation of WNT signaling in BALO by treatment with mo142-3p leads to branching defects without affecting cellular viability

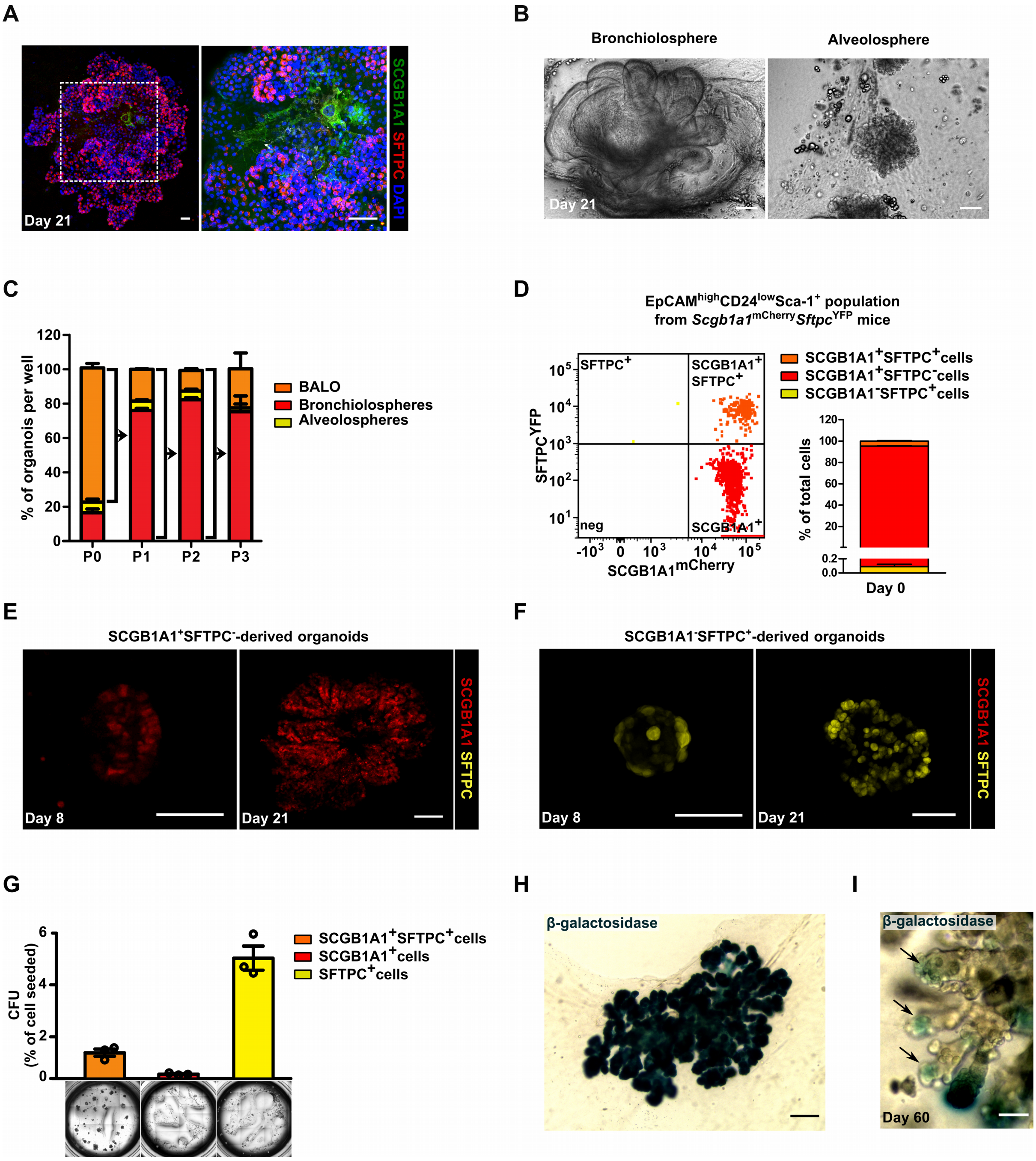

Appendix Figure S1. Initial culture of BASC with rMC primarily gives rise to BALO with a minor proportion of bronchiolospheres and alveolospheres formation

A. Representative fluorescence images of day 21 BALO section stained for the cell club marker SCGB1A1 and the AEC II marker SFTPC. Scale bars represent 50 (left) and 25 µm (right).

B. Representative images of a bronchiolosphere and alveolosphere present in EpCAM^{high}CD24^{low}Sca-1^{+} cells /rMC Matrigel co-cultures.

C. Percentages of BALO, bronchiolospheres and alveolospheres per well at P0 and P1 (only BALO), P2, and P3 in n=4 biological replicates.

D. Representative flow cytometric data- and percentages- of the composition of flow-sorted $EpCAM^{high}CD24^{low}Sca\text{-}1^{+}$ cell fractions (defined in Fig. 1A) calculated from the lungs of adult $\mathit{Scgb1a1}^{mCherry}\mathit{Sftpc}^{YFP}$ reporter mice according to mCherry and YFP expression in n=4 biological replicates.

E-F. Representative confocal images of days 8 to 21 of culture showing endogenous SCGB1A1 and SFTPC expression during bronchiolosphere (E) and alveolospheres (F) derived from $EpCAM^{high}CD24^{low}Sca\text{-}1^{+}$ cells sorted based on single expression of SCGB1A1 or SFTPC, respectively.

G. Colony forming units (CFU) from isolated $SFTPC^{+}$, $SCGB1A1^{+}$ and $SFTPC^{+}SCGB1A1^{+}$ cells cultured with rMC derived from the whole lung homogenate of $\mathit{Scgb1a1}^{mCherry}\mathit{Sftpc}^{YFP}$ reporter mice in n=3 biological replicates. Depicted are representative whole well pictures of day 21 co-cultures.

H. Representative image of β-galactosidase staining of BALO derived from the lungs of BASC v-race mice.

I. Representative image of β-galactosidase staining of BASC ($SCGB1A1^{+}SFTPC^{+}$) within the distal regions (arrows) of day 60 BALO derived from the lungs of BASC viewer mice.

Data information: Scale bars=100 µm (B, E-I). Bar charts presented as the mean ± S.E.M.

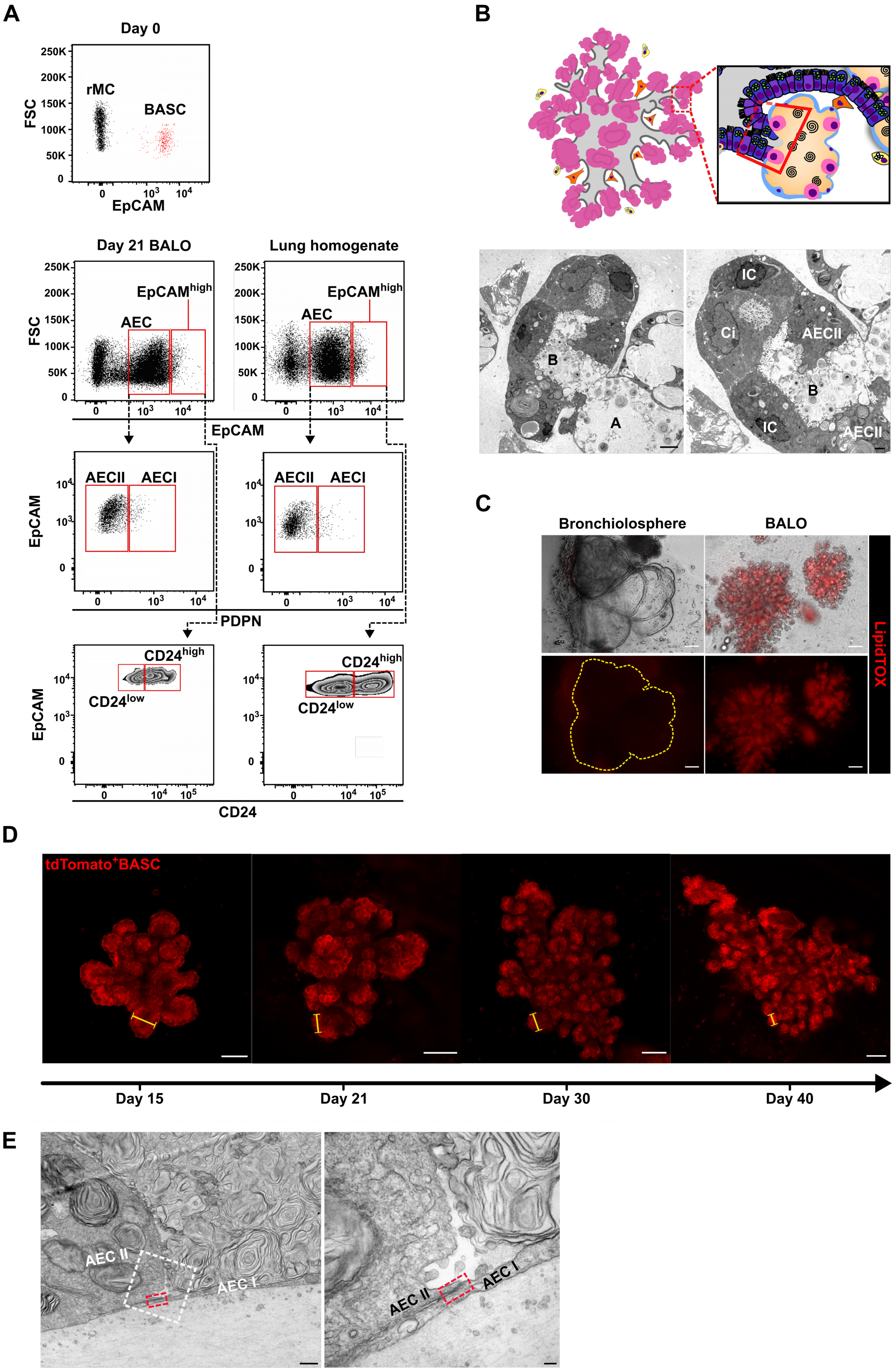
A
Day 0
FSC
EpCAM
rMC
BASC
Day 21 BALO
Lung homogenate
AEC
EpCAMhigh
AECII
AECI
PDPN
CD24high
CD24low
CD24
B
B
A
IC
Ci
AECII
C
Bronchiolosphere
BALO
LipidTOX
D
tdTomato+BASC
Day 15
Day 21
Day 30
Day 40
E
AEC II
AEC I

Appendix Figure S2. The BALO system resembles the cellular composition and organization of the bronchioalveolar compartment

A. Representative flow cytometric data of freshly sorted BASC population/rMC (day 0), lung homogenate, and cells isolated from day 21 BALO demonstrating expression of EpCAM, PDPN, and CD24.

B. Representative scheme and pictures of junction of tubular bronchiolus (B) followed by alveolar space (A) with flattened epithelial lining. A ciliated cell (Ci) is flanked on both sides by an intermediate cell (IC) type with structural criteria of AEC II but almost no lamellar bodies, followed by two typical AEC II (AEC II) with numerous lamellar bodies. Scale bars indicate 2500 nm (left) and 1000 nm (right).

C. Phospholipid staining of day 21 BALO and bronchiolosphere (outline marked in yellow) with the LipidTOX™ red phospholipidosis detection reagent. Scale bars represent 100 μm.

D. Representative confocal images of the alveolar diameters (yellow line) of alveoli in BALO at days 15, 21, 30 and 40 of culture. Scale bars represent 50 μm.

E. Electron microscopy of BALO alveoli showing a cuboidal AEC II and a thin elongated AEC I connected by tight junctions (red square). Scale bars indicate 500 nm (left) and 100 nm (right).

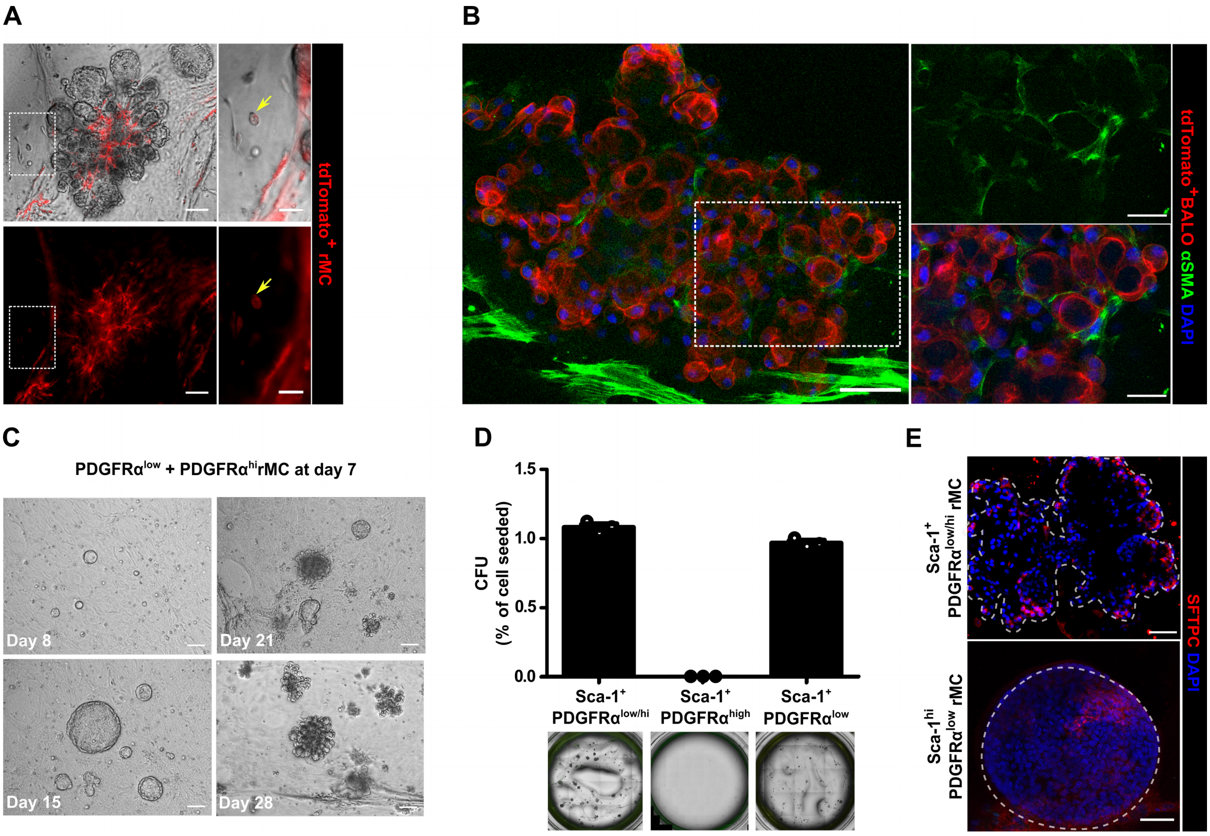

Appendix Figure S3. Defined rMC subsets contribute to BALO formation

A. rMC derived from mTmG mice at day 21 BALO. LIF in close proximity to BALO is depicted with a yellow arrow.

B. Fluorescence confocal images of αSMA staining within tdTomato$^+$ BALO cultures.

C. Representative images of BALO formation at day 8, to 28 of co-culture. WT BASC population was initially co-cultivated with only rMC expressing low of PDGFRα-GFP while rMC expressing high of PDGFRα-GFP were added at day 7 of culture.

D. CFU from WT BASC co-cultivated with sorted rMC (total PDGFRα$^+$ population) or rMC expressing either low or high levels of PDGFRα-GFP in n=3 biological replicates. Depicted are representative whole well pictures of day 21 co-cultures.

E. Representative images of BALO derived from WT BASC co-cultivated with sorted rMC (total PDGFRα$^+$ population) or rMC expressing either low levels of PDGFRα-GFP and stained with SFTPC.

Data information: Scale bars=100 µm (A, B, D) or 50 µm (C left, E) or 25 µm (C right). Bar charts presented as the mean ± S.E.M.

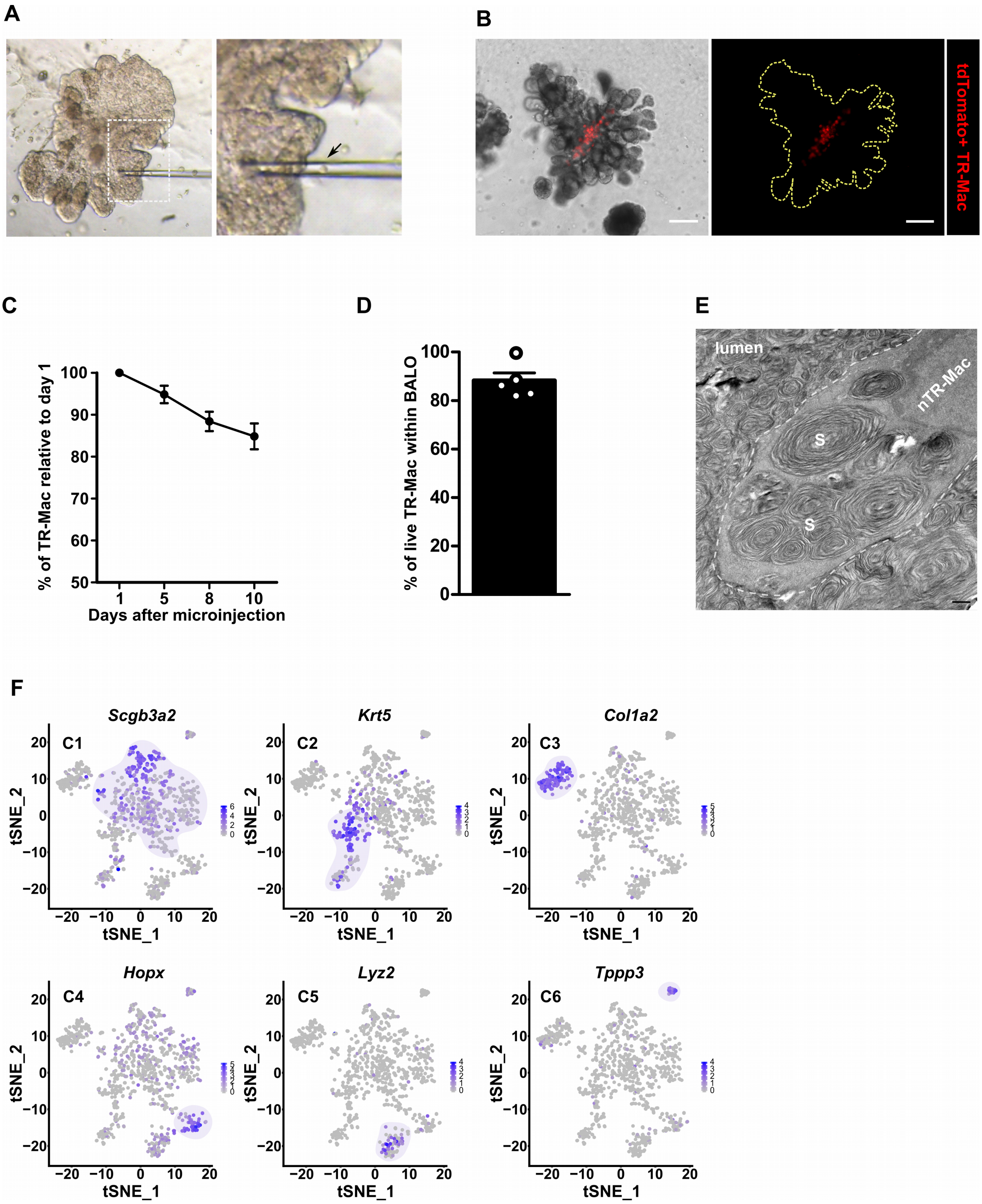

Appendix Figure S4. Tissue-resident macrophages engraft into BALO

A. Representative images of BALO puncture showing TR-Mac (arrow) in the microinjection needle.

B. Representative images of developing BALO directly after microinjection of $tdTomato^{+}$ TR-Mac into the central airway-like structures.

C. Percentage of TR-Mac survival over time after microinjection into BALO (n=5 biological replicates).

D. Percentage of live TR-Mac after 10 days post-microinjection into BALO (n= 5 BALO).

E. Representative EM image of surfactant digestion (S) by a TR-Mac showing an intact nuclear membrane in the lumen of BALO´s alveolar-like structures. nTR-Mac, TR-Mac nucleus.

F. tSNE plots of the digested day 23 BALO cultures depicting clusters: C1, club/secretory cells, *Scgb3a2*; C2, basal cells, *Krt5*; C3, rMC, *Col1a2*; C4, AEC I, *Hopx*; C5, AEC II, *Lyz2*; and C6, ciliated cells, *Tppp3*.

Data information: Scale bars=100 µm (B) or 500 nm (E). Bar charts presented as the mean ± S.E.M.

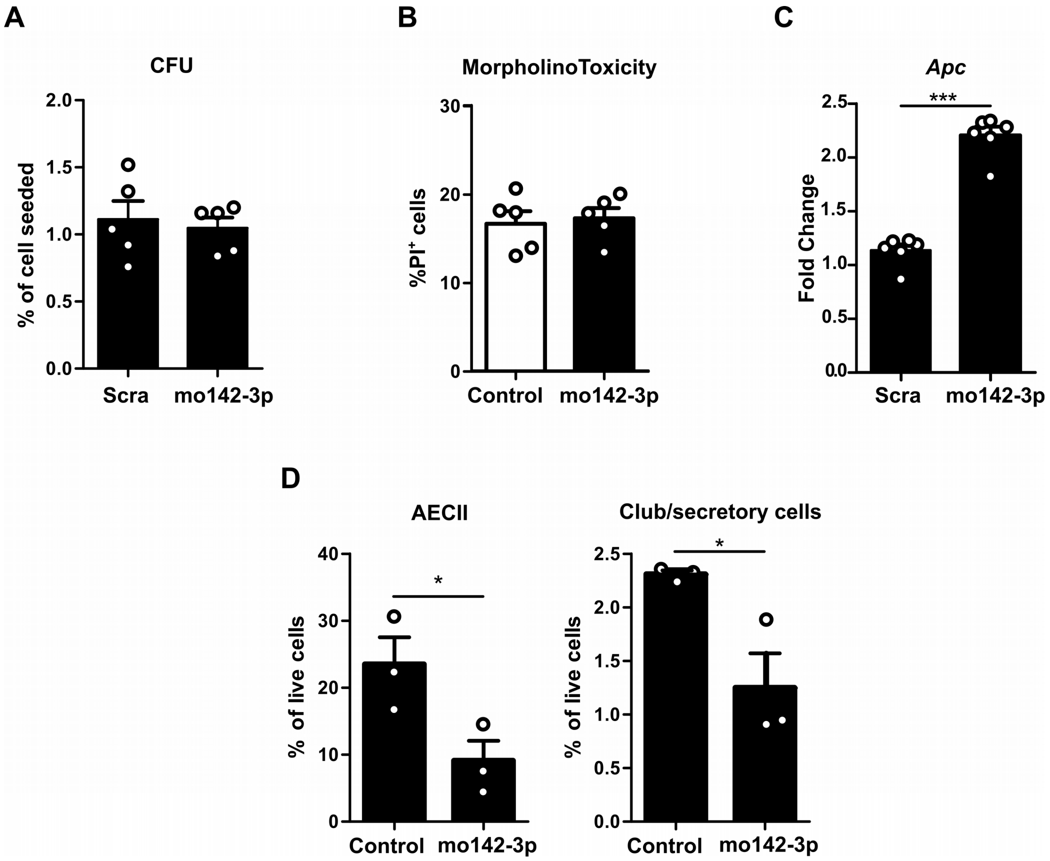

Appendix Figure S5. Downregulation of WNT signaling in BALO by treatment with mo142-3p leads to branching defects without affecting cellular viability

A. CFU in Scra- and mo142-3p-treated organoids 15 days after treatment (n=5 biological replicates).

B. Propidium iodide (PI) staining of BALO in untreated controls or after five-day treatment with mo142-3p (n=5 biological replicates).

C. mRNA levels of *Apc* expression in Scra and mo142-3p-treated organoids 5 days after treatment (n=6 biological replicates with pooled cells from 2 cultures per replicate.

D. Representative flow cytometric data showing the percentage of AEC II ($EpCAM^{+}CD49f^{int}Lyzotracker^{+}$) and club/ secretory cells ($EpCAM^{+}CD49f^{high}CD24^{low}$) in Scra and mo142-3p-treated organoids in n=3 biological replicates with pooled cells from 2 cultures per replicate.

Data information: Bar charts presented as the mean ± S.E.M. and probability determined using t.test (*P<0.05,***P<0.001).
