# Supplementary material for: Multilineage murine stem cells generate complex organoids to model distal lung development and disease
Source: EMBO J. 2020 Sep 28;39(21):e103476. doi: 10.15252/embj.2019103476 (PMC7604576; doi:10.15252/embj.2019103476)
Supplement: Supplementary file 2 — Expanded View Figures PDF [file EMBJ-39-e103476-s002.pdf]

Expanded View Figures

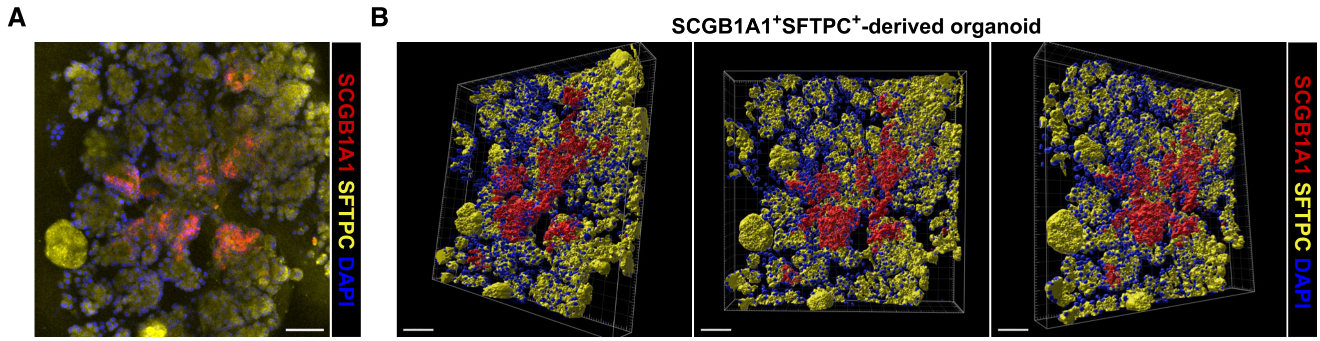

Figure EV1. 3D reconstruction of BALO depicts proximo-distal cell specification.

A, B Representative confocal picture (A) and 3D reconstruction (B) of a day 21 culture showing endogenous SCGB1A1 and SFTPC expression within BALO derived from EpCAM^{high}CD24^{low}Sca-1$^+$SCGB1A1$^+$SFTPC$^+$ cells isolated from *Scgb1a1*^{mCherry}*Sftpc*^{YFP} reporter mice. Scale bars represent 50 μm.

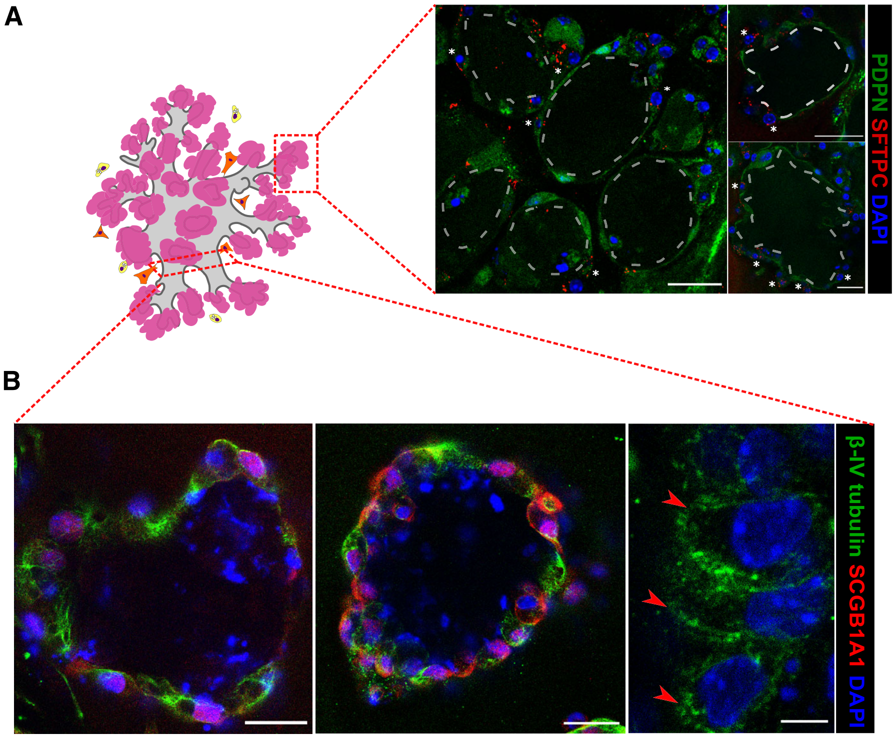

Figure EV2. BALOs express markers of differentiated airways and alveoli.

A, B Representative scheme and confocal images of the BALO alveolar-like (A) and airway-like (B) structures. (A) Representative image of AEC II SFTPC staining (white asterisks) and AEC I endogenous PDPN expression in day 28 BALO isolated from the lungs of *Pdpn*^{GFP} reporter mice. Scale bars represent 25 μm. Dotted lines indicate the alveoli. (B) Representative fluorescence images of BALO's airway-like structures stained for β-IV tubulin$^+$ ciliated cells (red arrowheads) and SCGB1A1$^+$ club cells. Scale bars represent 25 μm (left and middle) and 5 μm (right).

© 2020 The Authors

© 2020 The Authors
